# Elevated Manganese Concentrations in Drinking Water May Be Beneficial for Fetal Survival

**DOI:** 10.1371/journal.pone.0074119

**Published:** 2013-09-16

**Authors:** Syed Moshfiqur Rahman, Agneta Åkesson, Maria Kippler, Margaretha Grandér, Jena Derakhshani Hamadani, Peter Kim Streatfield, Lars-Åke Persson, Shams El Arifeen, Marie Vahter

**Affiliations:** 1 Institute of Environmental Medicine, Karolinska Institutet, Stockholm, Sweden; 2 International Centre for Diarrhoeal Disease Research, Bangladesh (icddr,b), Dhaka, Bangladesh; 3 International Maternal and Child Health, Department of Women’s and Children’s Health, Uppsala University, Uppsala, Sweden; Tabriz University of Medical Sciences, Islamic Republic of Iran

## Abstract

**Background:**

Elevated exposure to the essential element manganese (Mn) can be toxic. Manganese concentrations in ground water vary considerably, and reported associations between Mn and early-life mortality and impaired development have raised concern. We assessed the effects of drinking water Mn exposure during pregnancy upon fetal and infant survival.

**Methods:**

In this population-based cohort study, we identified the outcomes of pregnancies registered between February 2002 and April 2003 in Matlab, Bangladesh. Using inductively coupled plasma mass spectrometry, we measured the concentrations of Mn and other elements in the pregnant women’s drinking water.

**Results:**

A total of 1,875 women were included in the analysis of spontaneous abortions (n=158) and 1,887 women in the perinatal mortality analysis (n=70). Water Mn ranged from 3.0–6,550 µg/L (median=217 µg/L). The adjusted odds ratio (OR) for spontaneous abortion was 0.65 (95% CI 0.43–0.99) in the highest water Mn tertile (median=1,292 µg/L) as compared to the lowest tertile (median=56 µg/L). The corresponding OR for perinatal mortality was 0.69 (95% CI 0.28–1.71), which increased to 0.78 (95% CI 0.29–2.08) after adjustment for BMI and place of delivery (home/health facility; n=1,648).

**Conclusions:**

Elevated water Mn concentrations during pregnancy appear protective for the fetus, particularly in undernourished women. This effect may be due to the element’s role in antioxidant defense.

## Introduction

Elevated concentrations of manganese (Mn) in ground water are prevalent worldwide [1,2]. Manganese is an essential element utilized by antioxidants, including superoxide dismutase (MnSOD), and by various enzymes important in the metabolism of carbohydrates and proteins. Although Mn-dependent enzymes are important during early life development [3], excess Mn exposure may cause neurotoxicity, as shown experimentally and in neonates given parenteral nutrition [1,3]. Recently, much research has focused on potentially toxic effects of Mn in environmentally-exposed children, especially those drinking water containing elevated Mn levels (see Roels et al. [4]).

Intestinal absorption of Mn occurs mainly via divalent metal transporter 1 (DMT1), which is up-regulated when iron stores are low and in pregnancy [5]. As a result, maternal blood Mn concentrations progressively increase during pregnancy [6,7]. Because Mn easily passes through the placenta, the elevated gastrointestinal absorption during pregnancy may lead to excess fetal exposure, and potentially toxicity, in highly exposed mothers [8-10]. A cross-sectional study in Bangladesh, which included 1,628 mothers who had given birth to 3,824 children, found that the odds ratio for infant mortality nearly doubled (OR=1.9, 95% CI 1.2–2.9) when drinking water Mn concentrations were greater than 0.4 mg/L [11]. This finding was supported by an ecological study involving 100 counties in North Carolina, which showed a positive association between drinking water Mn and infant mortality [12]. However, another ecological study, carried out in several districts in Bangladesh, found no association between water Mn concentrations (based on the mean Mn concentration of 7–14 wells in each of 12 study areas; range: 0.03–1.58 mg/L) and registered infant deaths (n=934), constituting 3% of the 29,744 births [13].

To the best of our knowledge, no prospective study has explored the association between drinking water Mn and pregnancy outcomes. Therefore, the aim of the present study was to prospectively assess the effects of water Mn concentrations during pregnancy on fetal and infant survival. This study was carried out in a rural area of Bangladesh, where more than 40% of drinking water wells contain more than 0.4 mg Mn/L [14].

## Materials and Methods

### Study Population

**Figure 1:**
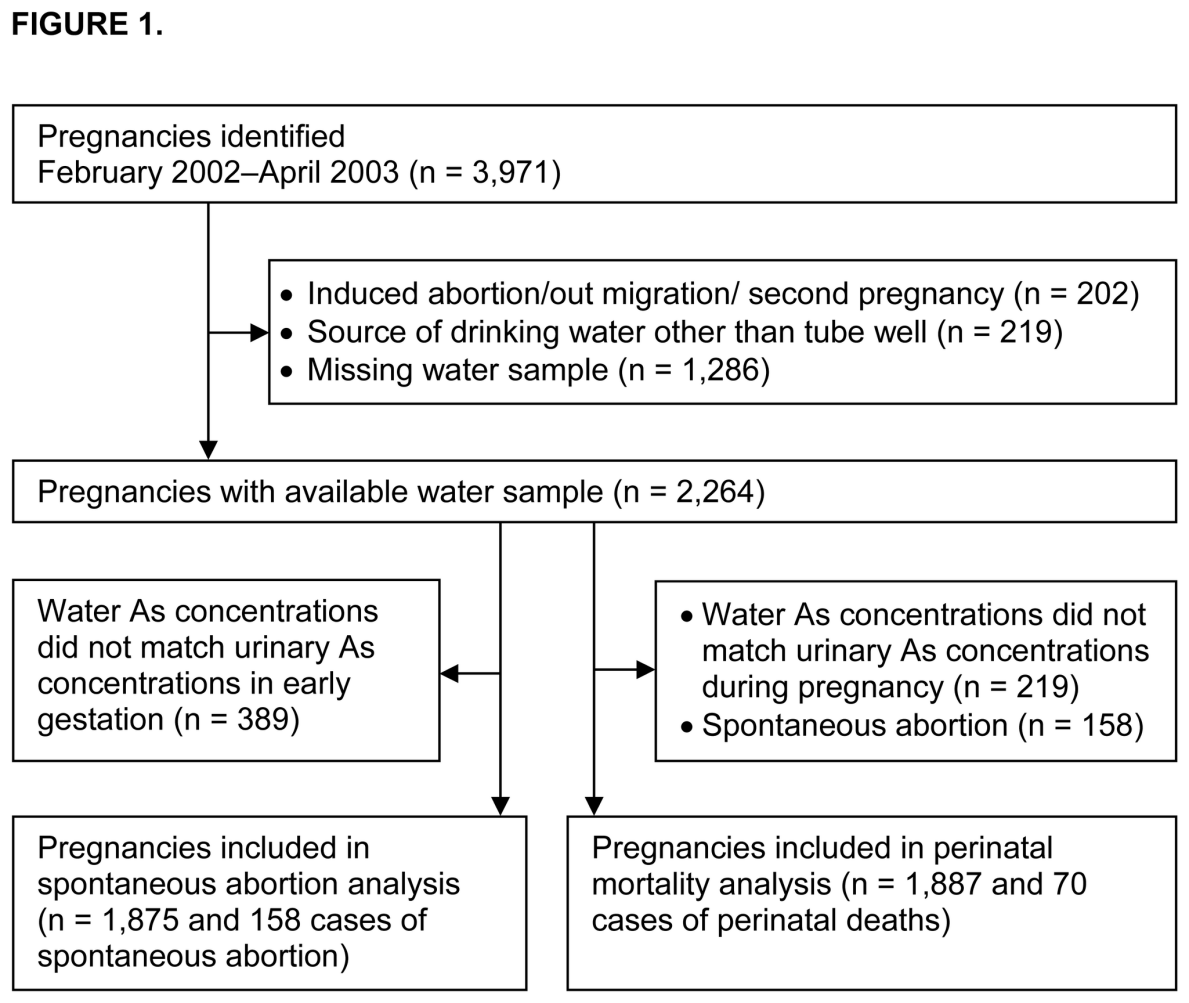
Flow chart depicting selection of pregnancy cohort (February 2002 to April 2003).

This study was part of our ongoing research on the consequences of environmental exposures early in life and was carried out in Matlab, a rural area of Bangladesh [15]. The International Center for Diarrhoeal Disease Research in Bangladesh (icddr,b) provides health services to the residents of the study area. It also has a well-established, population-based Health and Demographic Surveillance System (HDSS) that has been ongoing since the 1960s [16]. Community health workers visit households on a monthly basis to collect information on vital events such as pregnancies, births, deaths, and morbidities. Based on this information, we identified a population-based cohort of 3,971 women, who were pregnant between February 2002 and April 2003 (Figure 1). Pregnancy was identified by a urine test, most often in gestational week (GW) 9 (5^th^–95^th^ percentiles=5–15 weeks). Exclusion criteria for the study included migration out of the area (n=42) and induced abortion (n=110). If women became pregnant twice during the study period (n=50), only the first pregnancy was included in the analysis. We also excluded women who were drinking surface water (n=219), as it is prone to microbial contamination and this could contribute to study outcomes. Thus, 3,550 pregnant women of the original 3,971 identified were eligible for the study.

### Ethics Statement

This study was approved by the ethical review committees of icddr,b, Bangladesh and the Karolinska Institutet, Sweden. Written consent was obtained from all subjects prior to enrollment in the initial study. Subjects were free to leave the study at any time.

### Assessment of Manganese and Other Elements in Drinking Water

Because there is no suitable biomarker of Mn exposure [14], drinking water Mn was used as the measure of exposure. The drinking water samples for this study were available from a parallel, population-based study (AsMat), which evaluated the association between well water arsenic (As) and the presence of skin lesions [17]. Information on lifetime drinking water sources had been collected for all inhabitants over 4 years of age via household interviews. This information was used to identify water samples for the women in our cohort.

Drinking water samples were available for 2,264 (64%) of the 3,550 eligible pregnant women (Figure 1), and they were drawn from a total of 1,480 wells. The two primary reasons for missing drinking water samples (n=1,286) were (1) a lack of information on the relevant water source, because the pregnancy was identified after completion of the household water survey; or (2) that water samples were inadvertently discarded after the initial analysis of As. Changes in water source during pregnancy were common in this cohort due to a Bangladeshi tradition in which particularly primiparous women move to their parents’ homes for delivery. In addition, some families may have changed their water source in response to the identification of elevated As levels in their wells [18]. To minimize exposure misclassification stemming from well switching, we compared previously measured concentrations of drinking water As with the concentrations of As metabolites in urine, a useful biomarker of ongoing As exposure, at approximately GWs 8 and 30 [19]. We excluded women from our study if water and urinary As concentrations differed b**y** more than 100 µg/L, which left 1,875 and 1,887 pregnancies with reliable exposure data for the assessment of association with fetal and infant survival, respectively (Figure 1). The correlation between As concentrations in drinking water and urine was strong in the women included in the study, both at GW 8 (Spearman’s correlation, r_s_=0.85) and GW 30 (r_s_=0.73).

Water samples were collected in 20-mL polyethylene vials after flushing the water with approximately 30 strokes of the pump. The vials contained 30 µL concentrated nitric acid (69% HNO_3,_ Suprapur, MERCK, Germany) to prevent precipitation of iron (Fe), Mn, and As. The samples were stored at -20°C in Matlab prior to being transported to the Karolinska Institutet, Sweden, for analysis of Mn and other elements, including As, Fe, magnesium (Mg), calcium (Ca), and zinc (Zn). Element concentrations were measured using inductively coupled plasma mass spectrometry (ICP-MS; Agilent 7500ce, Agilent Technologies, Tokyo, Japan) with the collision cell in helium mode [14]. Before analysis, 1% HNO_3_ was added to the water samples at a ratio of 1:10. Analytical performance was ascertained by analysis of certified reference material (NIST 1643e, Trace Elements in Water, National Institute of Standards and Technology, Gaithersburg, MD), the results of which showed good agreement with the reference value.

### Assessment of Outcomes

Information on all pregnancy outcomes (spontaneous abortion, induced abortion, stillbirth, and live birth) and infant deaths was obtained from the HDSS. We evaluated the potential effect of water Mn concentrations on spontaneous abortion and perinatal mortality. Spontaneous abortion was defined as unintended loss of the fetus before GW 28. Thus, no form of induced abortion was included. Perinatal deaths included both stillbirths and early neonatal deaths. Stillbirth was defined as the death of a fetus at 28 completed weeks of gestation or later. Early neonatal death was defined as the death of an infant within one week (seven completed days) after birth. The denominator used for calculating the rate of spontaneous abortion was the total number of pregnancies, whereas for perinatal mortality the denominator was the total number of live births and births of dead fetuses after GW 28.

Information on the cause of infant death was based on a verbal autopsy questionnaire developed by the World Health Organization (WHO) and adapted for the Matlab HDSS area [20]. Interviews were conducted 2–6 weeks after the death of an infant, and they were administered to a caretaker or relative who had lived with the infant during the terminal stage of illness or death. Three physicians independently reviewed the questionnaires to assign cause of death. Cause of death was coded based on the tenth revision of the International Classification of Disease, Disease Injuries and Cause of Death (ICD-10).

### Assessment of Covariates

Information on women’s demographic characteristics (age, weight, height, date of birth, gravidity, education, and socioeconomic status) as well as place of delivery (home/health facility), infants’ sex and birth weight were available from the HDSS and from a parallel, population-based, randomized food and micronutrient supplementation trial (MINIMat) [21]. Maternal body-mass index (BMI), calculated based on women’s weight and height (kg/m^2^) at approximately GW 9, was used as a continuous variable. Gravidity was defined as the number of pregnancies a woman had experienced, including the present one. Maternal education was defined as the number of years of formal schooling. Socioeconomic status (SES) was based on a wealth index constructed from information on family ownership of a number of consumer items, construction type of the house, and dwelling characteristics (range: -5 to +5) [22]. It was used as a continuous variable. Gestational age in weeks was calculated by subtracting the date of the last menstrual period from the date of the pregnancy outcome. In the MINIMat trial, women were randomly allocated to two food groups and three micronutrient groups, the latter being initiated at GW 14 [21]. Because most of the women in the current sample participated in this trial, we tested if the supplementation influenced the analysis of perinatal outcome. Because marked seasonal differences in neonatal mortality have been reported in the study area, peaking in November [23], we included season of birth as a covariate, categorized as pre-monsoon (January–May), monsoon (June–September), or post-monsoon (October–December).

We assessed water concentrations of other essential or toxic elements (As, Fe, Zn, Ca, and Mg). Arsenic exposure has previously been shown to be associated with pregnancy outcomes in this area [15,24]. Water concentrations of the essential elements Fe, Zn, Ca, and Mg have also been associated with fetal growth and infant survival [25,26]. Finally, Fe in drinking water has been found to affect Fe status in another rural area in Bangladesh [27].

### Statistical Analysis

We used analysis of variance (ANOVA) and χ^2^ tests to assess differences in continuous and categorical variables, respectively, and Spearman’s rank correlation coefficients (r_s_) to assess associations between continuous variables. We used multivariable-adjusted logistic regression models to estimate odds ratios (ORs) and 95% confidence intervals (CIs) of spontaneous abortion and perinatal mortality in relation to tertiles of water Mn concentrations. The lowest tertile of exposure was used as the reference. In addition, we estimated ORs after assessing Mn concentrations (mg/L) as a continuous variable. Potential trends were evaluated using the slope of the appropriate adjusted logistic regression model.

Models were adjusted for variables that were known or proposed risk factors for pregnancy outcomes, were significantly associated with both exposure and outcome (*P* <0.05), or changed effect size estimates by 10% or more. We assessed collinearity between covariates, and variables that were strongly correlated (r_s_ >0.70) were not included in the same model. In addition, we stratified all analyses by BMI 18.5 kg/m^2^, the WHO cut-off for undernutrition [28]. Missing data on covariates were handled by complete subject analysis.

All tests were two-sided. Statistical significance was defined as a 95% CI that did not include zero or a *P* value < 0.05. Statistical analyses were conducted using SPSS (version 20.0, IBM Corporation, USA) and STATA (version 11, STATA Corp, College Station, TX, USA).

## Results

### Demographic Characteristics

There were no major differences between the spontaneous abortion (Table 1) and perinatal death (Table 2) cohorts in terms of demographic characteristics or drinking water concentrations of elements. Approximately 25% of the women in both cohorts were primigravida, and 50% of the women had 1–2 children. About 31% of the women delivered at health facilities. Only two women reported smoking during pregnancy, and none used alcohol. In both cohorts, women with missing water samples were slightly younger (mean ±SD age: 26±6 years), were more educated (6±4 years at school), had fewer children (1.2±1.4 children), and were more often pregnant for the first time (36%) than women with water samples.

**Table 1:** Demographic characteristics of women included in the analysis of spontaneous abortion, by water manganese (Mn) tertiles.

**Variables** ^1^	**No of women**	**Tertiles of Mn in drinking water (µg/L**)			
		**<103**	**103–599**	**>599**	***P*-value^2^**
Age (years)	1,875	27.1±6.2	26.8±6.0	27.2±6.1	0.470
Weight (Kg)	1,699	45.1±6.4	45.1±6.6	46.2±7.3	0.007
Height (cm)	1,699	150.0±5.2	149.9±5.2	149.9±5.2	0.813
BMI (kg/m^2^)	1,699	19.9±2.5	20.0±2.6	20.4±2.9	0.001
Gravidity	1,873	2.8±1.6	2.8±1.6	2.8±1.7	0.711
Education (years)	1,819	4.4±3.8	4.7±3.9	5.2±4.0	0.002
Socioeconomic status (SES)^3^	1,830	-0.3±2.2	-0.1±2.3	0.2±2.2	0.002
Well depth (m)	1,329	44.6±42.9	44.7±35.4	56.8±23.4	<0.001
Drinking water concentrations^4^					
Mn (µg/L)	1,875	56 (17–97)	219 (110–555)	1292 (657–3188)	<0.001
As (µg/L)	1,875	117 (2.6–303)	115 (0.3–518)	0.7 (0.1–425)	<0.001
Fe (mg/L)	1,875	2.8 (0.7–5.9)	4.9 (0.3–13)	0.4 (0.1–9.4)	<0.001
Mg (mg/L)	1,875	21 (5.5–46)	19 (4.9–48)	19 (8.9–38)	0.626
Ca (mg/L)	1,875	31 (8.4–58)	43 (15–100)	52 (22–107)	<0.001
Zn (µg/L)	1,875	15 (1.8–133)	17 (2.6–121)	21 (2.9–240)	0.474

^1^Data shown as mean ± SD or median (5^th^–95^th^ percentile)

^2^Derived by ANOVA

^3^SES: based on a number of wealth indices (range= -5 to + 5 )

^4^Drinking water used in early pregnancy

**Table 2:** Demographic characteristics of women included in the analysis of perinatal mortality, by water manganese (Mn) tertiles.

**Variables** ^1^	**No of women**	**Tertiles of Mn in drinking water (µg/L**)			***P*-value** ^2^
		**<103**	**103–621**	**>621**	
Age (years)	1,887	26.8±6.0	26.6±5.8	27.1±6.1	0.378
Weight (kg)	1,756	44.9±6.2	44.9±6.7	46.1±7.2	0.001
Height (cm)	1,756	150.1±5.2	149.8±5.3	149.9±5.1	0.671
BMI (kg/m^2^)	1,756	19.9±2.5	20.0±2.6	20.5±2.9	<0.001
Gestational age at birth (week)	1,875	39.1±2.4	38.9±2.6	38.9±2.7	0.554
Preterm delivery (% <37 weeks)	242	11.0	14.7	12.9	0.149
Gravidity	1,885	2.8±1.6	2.8±1.6	2.8±1.7	0.892
Education (years)	1,827	4.5±3.8	4.8±3.9	5.2±4.0	0.003
Socioeconomic status (SES)^3^	1,850	-0.3±2.2	-0.2±2.4	0.1±2.2	0.001
Birth weight (g) of live births	1,519	2,707±383	2,670±388	2,696±415	0.307
Season of birth (%)^4^					
Pre-monsoon	596	28.3	33.1	33.3	
Monsoon	574	28.2	31.6	31.4	0.017
Post-monsoon	717	43.5	35.3	35.2	
Delivery at health facilities (%)	694	38.0	38.0	39.2	0.827
Well depth (m)	1,348	46±45	46±38	57±23	<0.001
Drinking water concentrations^5^					
Mn (µg/L)	1,887	55 (17–97)	221 (109-583)	1,341 (667–3,338)	<0.001
As (µg/L)	1,887	112 (2.6–306)	112 (0.3–513)	0.6 (0.1–405)	<0.001
Fe (mg/L)	1,887	2.7 (0.7–5.9)	4.8 (0.3–13)	0.4 (0.1–8.4)	<0.001
Mg (mg/L)	1,887	20 (4.9–46)	19 (4.9–48)	19 (8.9–38)	0.980
Ca (mg/L)	1,887	30 (7.9–58)	42 (14–99)	52 (22–105)	<0.001
Zn (µg/L)	1,887	14 (1.6–131)	17 (2.6–115)	20 (2.5–240)	0.421

^1^Data shown as mean ± SD or median (5^th^–95^th^ percentile)

^2^Derived by using ANOVA or the χ^2^ test

^3^SES: based on a number of wealth indices (range= -5 to + 5 )

^4^re-monsoon (January–May), monsoon (June–September), and post-monsoon (October–December)

^5^Drinking water used during pregnancy

Overall mean and median concentrations of drinking water Mn were 615 µg/L and 217 µg/L, respectively, with the 5^th^ and 95^th^ percentiles being 28 µg/L and 2,326 µg/L (total range: 3–6,550 µg/L). Water Mn concentrations were inversely correlated with water concentrations of As (r_s_=-0.46) and Fe (r_s_=-0.25) and positively correlated with water Ca concentrations (r_s_ =0.49), but they were not significantly correlated with water Zn (r_s_=0.15) or Mg (r_s_=0.07) concentrations. Water Mn concentrations were higher in deeper wells (those >50 m; median=734 µg/L) than in shallow wells (those <50 m; median=126 µg/L). By contrast, As and Fe concentrations were higher in shallow wells, which had medians of 193 µg/L and 3.9 mg/L, respectively, than in deeper wells, which had medians of 2.6 µg/L and 1.2 mg/L, respectively. The concentrations of Ca, Zn, and Mg did not vary by the depth of the well; overall median concentrations were 42 mg/L, 18µg/L, and 20 mg/L, respectively.

### Spontaneous Abortion

Of the 1,875 pregnancies included in the analysis, 158 (8.4%) resulted in spontaneous abortion, which occurred, on average, in GW 12 (range: GWs 5–27). Compared with the women included in the cohort, women with missing water samples or discordant water and urine As concentrations had a slightly higher frequency of spontaneous abortion (8.4% vs. 11%; *P*=0.005).

As shown in Table 1, women in the highest water Mn tertile had higher BMI, SES, and water Ca concentrations; were more educated; and had lower water As and Fe concentrations than women in the lowest Mn tertile. The adjusted OR for spontaneous abortion (Table 3) was significantly lower for women in the highest Mn tertile (median Mn=1,292 µg/L, OR=0.65, 95% CI 0.43–0.99; model I) compared with the women in the lowest tertile (median Mn=56 µg/L). Additional adjustment for BMI, SES, and mother’s education (model II), which reduced the sample size to 1,642, did not change the estimate (OR=0.66, 95% CI 0.40–1.10) for the highest Mn tertile. Using water Mn concentrations as a continuous variable, the adjusted OR was 0.75 (95% CI 0.58–0.98, model I) per 1 mg/L (1,000 µg/L) increase in Mn concentration, with a significant trend (*P*=0.034). Additional adjustment (model II) changed the OR only marginally (0.78, 95% CI 0.57–1.06) per 1 mg/L increase in Mn concentration; *P* for trend=0.109.

**Table 3:** Adjusted logistic regression analysis of the association between water manganese (Mn) concentrations and spontaneous abortion.

	**No pregnancies**	**No spontaneous abortions**	**Tertiles of drinking water Mn concentrations (µg/L**)		
			<103^1^	**103–599**	**>599**
Crude OR (95% CI)	1,875	158	1.00	0.82 (0.56–1.20)	0.64 (0.42–0.96)
OR for multivariable-adjusted model I^2^ (95% CI)	1,873	158	1.00	0.77 (0.51–1.16)	0.65 (0.43–0.99)
OR for multivariable-adjusted model II^3^ (95% CI)	1,642	102	1.00	0.69 (0.41–1.16)	0.66 (0.40–1.10)

^1^Reference category

^2^Model I: OR (95% CI) adjusted for gravidity and drinking water As and Fe concentrations

^3^Model II: also adjusted for maternal education, BMI, and SES

We did not adjust for maternal age due to its collinearity with gravidity; however, including the age of the mother instead of gravidity in the adjusted model gave similar results. Stratifying by BMI gave a model II-adjusted OR of 0.61 (95% CI 0.27–1.35) per 1 mg/L increase in water Mn for undernourished women (BMI <18.5; n=460) and an OR of 0.83 (95% CI 0.60–1.15) for women with BMI ≥18.5 kg/m^2^ (n=1,182). We performed a separate analysis of spontaneous abortion in relation to gestational age (with a median split at GW 12) and obtained a model II-adjusted OR of 0.64 (95% CI 0.37–1.09) per 1 mg/L increase in drinking water Mn before GW 12 and an OR of 0.88 (95% CI 0.61–1.27) thereafter.

### Perinatal Mortality

The 1,887 pregnancies included in the perinatal cohort resulted in 37 (2%) stillbirths and 1,850 (98%) live births. The mean gestational age at stillbirth and live birth was 36 weeks (range: 29–42) and 39 weeks (range: 23–47), respectively. Of the total 47 infant deaths during the first year of life, two-thirds (n=33) were early neonatal deaths, in accordance with another study conducted in the same geographical area [20]. Causes of early neonatal death included birth asphyxia (n=14), infections (n=7), complications of prematurity or low birth weight (n=8), congenital defects (n=3), and unspecified causes (n=1). Hence, 70 perinatal deaths, consisting of 37 stillbirths and 33 early neonatal deaths, were included in the analysis. Compared with the women included in the cohort, women with missing water samples or discordant water and urine As concentrations had similar rates of perinatal mortality (3.7% vs. 4.5%, respectively; *P*=0.233). As expected, the risk of perinatal mortality was significantly higher in women with preterm delivery (GW <37; n=242) than those who delivered at term (OR 7.72, 95% CI 4.73–12.61).

As shown in Table 2, women in the highest water Mn tertile had higher BMI, were more educated with higher SES, had higher water Ca concentrations, and had lower As and Fe concentrations than women in the lowest water Mn tertile. In the lowest Mn tertile, there were more deliveries after the monsoon than in other seasons or in other Mn tertiles (*P*=0.017). No significant association was observed between Mn concentrations in drinking water and perinatal mortality in an adjusted regression analysis (Table 4). Although lower odds of perinatal mortality (OR=0.69, 95% CI 0.28–1.71; model I) were observed among women in the highest water Mn tertile (median Mn=1,341 µg/L) than women in the lowest tertile (median Mn=55 µg/L), the OR estimate changed by 13% (from 0.69 to 0.78) after adjustment for BMI and place of delivery (model II; n=1,648). We did not include gravidity as a covariate because of collinearity with maternal age; however, we found similar results when we included gravidity rather than maternal age in the model.

**Table 4:** Adjusted logistic regression analysis of the association between water manganese (Mn) concentrations and perinatal mortality.

	**No pregnancies**	**No perinatal deaths**	**Tertiles of drinking water Mn concentrations (µg/L**)		
			<103^1^	**103–621**	**>621**
Crude OR (95% CI)	1,887	70	1.00	1.14 (0.64–2.04)	1.04 (0.58–1.89)
OR for multivariable adjusted model I^2^ (95% CI)	1,779	67	1.00	0.99 (0.49–1.98)	0.69 (0.28–1.71)
OR for multivariable-adjusted model II^3^ (95% CI)	1,648	57	1.00	1.08 (0.50–2.33)	0.78 (0.29–2.08)

^1^Reference category

^2^Model I: OR adjusted for gestational age at birth, maternal age, education, SES, season at birth, and concentrations of As, Fe, Mg, Ca, and Zn in drinking water

^3^Model II: also adjusted for BMI and place of delivery

## Discussion

This prospective cohort study in rural Bangladesh did not detect any increased risk of spontaneous abortion or perinatal mortality with increasing drinking water Mn concentrations. On the contrary, women in the highest tertile of water Mn concentrations (median=1,292 µg/L) had an approximately 35% reduced risk of spontaneous abortion compared with women in the lowest tertile (median=56 µg/L). This estimated effect did not change significantly after adjustment for important covariates, including BMI and drinking water As concentrations.

Only two previous epidemiological studies, both ecological in design, have assessed the association between water Mn concentrations and pregnancy outcomes. These studies found no difference in the Mn concentrations of community drinking water between hospital cases of spontaneous abortion (n=286) and live births (n=1,391) [29] or between cases of stillbirth (n=77) and matched controls [30]. However, there are reasons to believe that exposure to Mn via drinking water can be beneficial particularly in early pregnancy, as indicated in the present cohort study. Placental oxidative stress in early pregnancy is one of the underlying mechanisms in the pathogenesis of spontaneous abortion [31]. Although embryonic organogenesis is largely anaerobic, there is a burst of oxidative stress in the mitochondria-rich placenta, particularly in the syncytiotrophoblast, at 8–12 weeks of pregnancy, when maternal blood flow to the placenta is established [32]. Therefore, adequate antioxidant defense is essential to protect the placenta from excessive oxidative stress and related tissue damage, which might lead to fetal loss. In addition to cytoplasmic Cu and Zn superoxide dismutase (Cu/ZnSOD), the mitochondrial MnSOD functions as an antioxidant for detoxifying superoxide anions in the placenta [33], and there is an increase in plasma MnSOD towards the end of the first trimester [32]. Thus, Mn in drinking water might improve the antioxidant capacity, particularly in undernourished women with prevalent Zn deficiency [34], as in the present study. Indeed, we observed a stronger protective association in women with low BMI.

Typically, food constitutes a major source of Mn, with cereals, rice, and vegetables representing especially important contributors. A Bangladeshi woman who consumes 300 g of rice (dry weight) daily [35], which in the present cohort contained on average 7.4 mg Mn/kg [36], would ingest approximately 2 mg Mn per day. This is thought to afford an adequate daily intake during pregnancy [37]. However, because the bioavailability of water Mn is likely to be higher than that of Mn ingested via a phytate-rich, rice-based diet [38], Mn-rich water might be an important source of absorbed Mn as well. Assuming a daily consumption of 3 L of water in this rural area of Bangladesh [35], half of the women will receive more than 0.6 mg Mn per day from drinking water, with those in the highest Mn tertile ingesting almost 4 mg/day via this route.

We did not find any clear evidence of an association between water Mn concentrations and perinatal mortality. We cannot directly compare our results with the retrospective study by Hafeman et al. [11], which reported a positive association between drinking water Mn and infant mortality in Bangladesh, as they did not report the age of the dead infants. Furthermore, they assessed a combination of several study cohorts selected for various reasons, including low As exposure. When Hafeman et al. considered only the 2,416 infants selected specifically for the evaluation of water Mn and infant mortality, adjusting for water As, they did not find any association. Thus, their results may not contrast with ours.

Strengths of this study include the population-based, prospective design; large sample size; early identification of pregnancy; and collection of outcome data within the well-established HDSS [16]. Exposure misclassification was minimized by validating the water sources used during pregnancy and by employing a reliable ICP-MS method to measure the water concentrations of Mn and other elements. For unknown reasons, women with missing water samples had a higher rate of spontaneous abortion, but not of perinatal mortality, compared to the women included in the study. However, as all women included in this prospective study had complete follow-up with respect to pregnancy outcome and the fate of their infants, this difference could not have had any major effect on the results.

In conclusion, it appears that elevated drinking water Mn concentrations during pregnancy may protect against fetal loss, possibly due to the role this essential element plays in antioxidant capacity. Whether the present findings are specific to undernourished Bangladeshi women or relevant to other populations requires further investigation. Potential adverse effects of exposure to Mn-rich water in early childhood, such as neurotoxicity, should be evaluated as well.
